# IDDI: integrated domain-domain interaction and protein interaction analysis system

**DOI:** 10.1186/1477-5956-10-S1-S9

**Published:** 2012-06-21

**Authors:** Yul Kim, Bumki Min, Gwan-Su Yi

**Affiliations:** 1 Department of Bio and Brain Engineering, KAIST, Daejeon 305-701, South Korea

## Abstract

**Background:**

Deciphering protein-protein interaction (PPI) in domain level enriches valuable information about binding mechanism and functional role of interacting proteins. The 3D structures of complex proteins are reliable source of domain-domain interaction (DDI) but the number of proven structures is very limited. Several resources for the computationally predicted DDI have been generated but they are scattered in various places and their prediction show erratic performances. A well-organized PPI and DDI analysis system integrating these data with fair scoring system is necessary.

**Method:**

We integrated three structure-based DDI datasets and twenty computationally predicted DDI datasets and constructed an interaction analysis system, named IDDI, which enables to browse protein and domain interactions with their relationships. To integrate heterogeneous DDI information, a novel scoring scheme is introduced to determine the reliability of DDI by considering the prediction scores of each DDI and the confidence levels of each prediction method in the datasets, and independencies between predicted datasets. In addition, we connected this DDI information to the comprehensive PPI information and developed a unified interface for the interaction analysis exploring interaction networks at both protein and domain level.

**Result:**

IDDI provides 204,705 DDIs among total 7,351 Pfam domains in the current version. The result presents that total number of DDIs is increased eight times more than that of previous studies. Due to the increment of data, 50.4% of PPIs could be correlated with DDIs which is more than twice of previous resources. Newly designed scoring scheme outperformed the previous system in its accuracy too. User interface of IDDI system provides interactive investigation of proteins and domains in interactions with interconnected way. A specific example is presented to show the efficiency of the systems to acquire the comprehensive information of target protein with PPI and DDI relationships. IDDI is freely available at http://pcode.kaist.ac.kr/iddi/.

## Background

Protein interactions, including binary PPIs and co-complexes, regulate biological process and biochemical reactions. Discovering protein interactions provides detailed interpretation of cellular mechanism of biological functions. Therefore, the identification of protein interaction is a critical issue for biology researchers. Recently, massive amount of protein interaction data is available due to the advancement of large-scale screening techniques such as yeast two-hybrid, affinity purification followed by mass spectrometry. Lots of protein interaction data verified from different experimental methods is publically available. However, although the increased data can give a landscape of the protein interactome, they are not much informative in detailed binding mechanisms and high false positive rate of the data is a big hurdle to interpret the interactome [1].

Investigating protein interactions in domain level can complement these limitations. Proteins consist of one or multiple domains thought as functional units of protein. In most cases, domain-domain interactions (DDIs) are crucial clues of protein interactions. Therefore, DDIs can be key supporting evidences for protein interaction mechanisms.

DDIs first have been identified based on 3-dimensional (3D) structures of protein complexes from Protein Data Bank [2]. 3DID [3], iPfam [4] and PInS [5] extract DDIs from the binding regions in known 3D structures. However, these datasets cover only a small proportion of DDIs due to insufficient available 3D structures. DDIs obtained from 3D structures cover less than 20% of the PPIs in Escherichea coli, Saccharomyces cerevisiae, Caenorhabditis elegans, Drosophila melanogaster and Homo sapiens [6]. To complement DDIs, various computational methods have been proposed to predict DDIs in recent years [7-25]. However, it is a cumbersome work for individual researchers to gather and integrate each predicted dataset because reliability of each datasets should be further analyzed since each method has different reliability level. Therefore, it is necessary to build an integrated system which combines all DDIs with a unified reliability scoring scheme.

Up to now, two combined DDI databases, DOMINE [26] and UniDomInt [27], have been published. DOMINE combined two 3D structure-based DDI datasets and thirteen predicted DDI datasets. Confidence level of each predicted DDIs in DOMINE is classified as High, Middle and Low based on the prediction overlap indexes (POIs) of the predicted DDI dataset. On the other hand, UniDomInt merged two 3D structure-based DDI datasets and eight predicted DDI datasets. UniDomInt provides numerical reliability scores for predicted DDIs by comparing an accuracy of the predicted datasets. Although DOMINE and UniDomInt provide a large amount of DDIs and compare the reliabilities between predicted DDIs with a unified format, some datasets are outdated and the total number of datasets is far below than that of currently published. They also ignored the scores measured by each prediction method of the datasets, so it is impossible to compare reliabilities between DDIs predicted in the same datasets. In addition, DOMINE and UniDomInt do not provide PPI information mediated by DDIs.

In this paper, we proposed an integrated analysis system for DDIs and their related protein interactions, called IDDI. We first combined three 3D structure-based DDI datasets and twenty predicted DDI datasets. To estimate the reliability of predicted DDIs, we developed a novel scoring scheme considering the individual accuracy of each datasets, independency among the datasets and the internal prediction scores of the DDIs measured by each method. Total amount of DDIs is increased significantly compared to previous comprehensive DDI databases, and the novel reliability scoring scheme achieved outstanding performance on sorting highly reliable DDIs. Furthermore, we joined our new DDI database with comprehensive PPI database, ComBiCom [28], and constructed a unified analysis system with a unique interface for the protein interaction network analysis that enables exploring the protein and domain interaction mechanism together.

## Methods

### Data sources

To construct a new comprehensive DDI database, we merged three 3D structure-based DDI datasets and twenty predicted DDI datasets based on the Pfam identifier. Since the datasets, including P-value, HiMAP, DomainGA and Top-down, use SCOP and InterPro identifier, we converted the SCOP domains to Pfam using SGD http://www.yeastgenome.org and the InterPro domains to Pfam using a mapping table in InterPro website http://www.ebi.ac.uk/interpro. Although other datasets used Pfam identifier, they are consisted with different versions of Pfam and it is susceptible to display same interactions differently since some domains were changed or eliminated as Pfam is updated. We therefore unified Pfam version of all datasets into the same release of Pfam-A 24.0 version. All domains which are not available at Pfam 24.0 were discarded. All combined DDI datasets with their number of DDIs and domains are listed in Table 1.

**Table 1:** Statistics and confidence scores of DDI datasets in IDDI

DDI Data [Ref.]		No. of Domains	No. of DDIs	Confidence Score
**3D-structure Based Datasets**	3DID [3]	4,233	6,039	
	IPfam [4]	2,935	4,119	
	PInS [5]	2,297	2,898	

	APMM[7]	2,082	14,023	0.1647
	DIMA-DPEA [8]	3,826	28,144	0.1020
	DIMA-DProf [8]	1,079	22,185	0.0475
	DIMA-String [8]	1,073	2,799	0.1975
	DIPD [9]	1,235	2,156	0.3924
	DomainGA [10]	148	275	0.1645
	DPEA [11]	1,022	1,811	0.2627
	GPE [12]	1,580	6,365	0.1805
	HiMAP [13]	206	257	0.2103
**Predicted Datasets**	InterDom [14]	5,546	144,793	0.1476
	IPPRI [15]	873	998	0.2177
	KGIDDI [16]	1,559	5,646	0.0513
	LLZ [17]	1,948	5,737	0.0915
	ME [18]	1,226	2,373	0.5929
	PE [19]	1,225	2,856	0.2348
	P-value [20]	398	596	0.1047
	RCDP [21]	484	960	0.2082
	RDFF [22]	616	2,413	0.0993
	Top-down [23]	4,303	22,221	0.3462
	TW [24]	165	170	0.4254

### Assessment of the reliability score for the predicted DDI

Each predicted DDI in our new database is evaluated by a reliability score. We considered three factors that affect reliabilities i) a confidence level of the each predicted dataset, ii) an independency of the dataset and iii) a local prediction score of the DDIs measured by each dataset.

#### Confidence score

Each predicted dataset has different confidence level. Predicted DDIs are more reliable when they were found in more accurate datasets.

To estimate the confidence levels, we used a weighted overlap method which measured a similarity between two datasets [27]. Weighted overlap (Wo) scores between each predicted dataset and a gold-standard positive (GSP) set could be a criterion of the confidence level. To prevent errors due to the difference of domains between two datasets, the weighted overlap method uses DDIs whose interacting domains were found in both datasets. For the two DDI datasets *a *and *b*, the Wo score is defined as:

$$Wo_{a,\;b}=\left(\frac{2\left(I_{a}\cap I_{b}\right)}{I_{a\to b}+I_{b\to a}}\right)$$

where I is a set of DDIs, *I*_*a*→*b *_is a subset of *I_a _*which interacting domains belong to both dataset a and b and, likewise, *I*_*b*→*a *_is a subset of *I*_*b *_which interacting domains are found in both datasets.

The GSP set were generated using 3D structure-based DDIs extracted from 3DID, iPfam and PInS. This GSP set contains total 6,768 verified DDIs. With the GSP set, the confidence score *C *of the predicted dataset *d *is defined as:

$$C_{d}=W{o_{d,}}_{GSP}$$

Table 1 shows confidence scores of each predicted dataset. Although the gap between two scores does not stand for absolute difference between two datasets, it is quite obvious that the DDIs are more reliable as they were predicted in higher confidence datasets. Based on confidence scores, the most reliable dataset is ME, followed TW, DIPD and Top-down. In contrast, RDFF, LLZ KGIDDI and DIMA-DPROF has low confidence scores which means DDIs predicted in these datasets have a high probability of false positive.

#### Independence score

**Figure 1:**
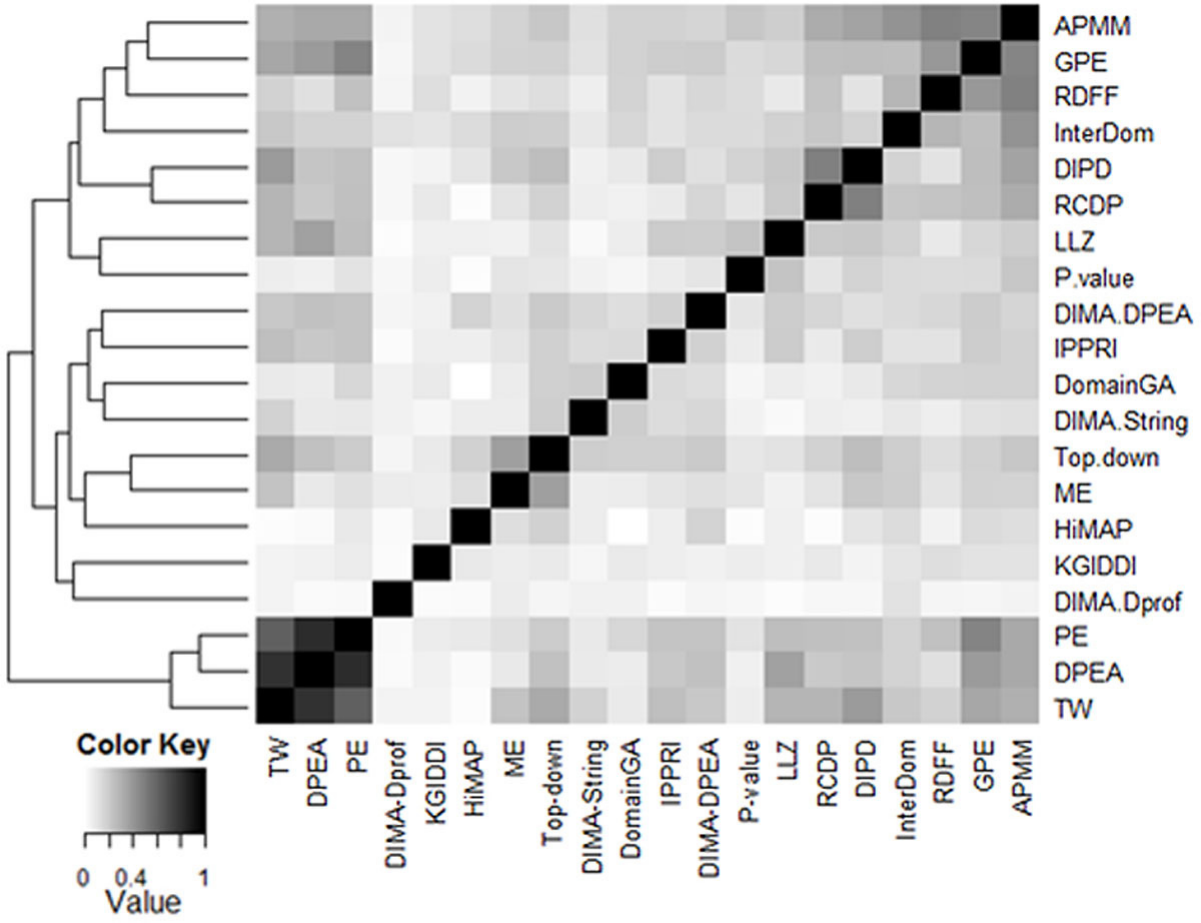
**Data similarity between each predicted dataset pair based on weighted overlap scores**.

Figure 1 shows unsupervised hierarchical clustering of the weighted overlap scores between every pairs of the datasets. The result reveals that DPEA and PE predicted quite similar DDIs because of their resembling prediction methodologies [26]. More than 95% of the DDIs predicted by DPEA are also found in PE and it causes overestimation problems for measuring reliability score of the DDIs.

We, therefore, considered how well the datasets that predict the same DDI are independent from each other for estimating reliabilities. For every dataset *d *that contain DDI *i*, the independence score *ID *is defined as:

$$ID_{d,\;\;i=}\frac{1}{1+\sum_{d\neq e}Wo_{d,\;\;e}}$$

where *e *is the all datasets that predict *i *except *d*. For example, a dataset whose DDI is not overlapped with other datasets will receive an independence score of one.

#### Prediction score

Local prediction scores of DDIs measured by each predicted dataset are also important key evidence for inferring reliabilities. Although DDIs were found in a same dataset, reliabilities of these DDIs are discrete depending on prediction scores. We scaled different ranges of original prediction score of each dataset from 0 to 1 by using an ordinal scaling method. Six of the datasets including HiMAP, KGIDDI, LLZ, RDFF, P-value and TW don`t provide own prediction scores. DDIs predicted in these datasets receive an average prediction score of the DDIs found in the same number of the datasets.

#### Reliability score

Using confidence scores, independence scores and prediction scores, we calculated a reliability score for each predicted DDI. For a predicted DDI *i*, a reliability score *R *is defined as:

$$R_{i}=\sum C_{d}\cdot ID_{d,i}\cdot P_{d,i}$$

where *d *is the all datasets that predicted *i *and *P_d, i _*is a prediction score of *i *measured by the dataset *d*.

### Integrated analysis system construction

**Figure 2:**
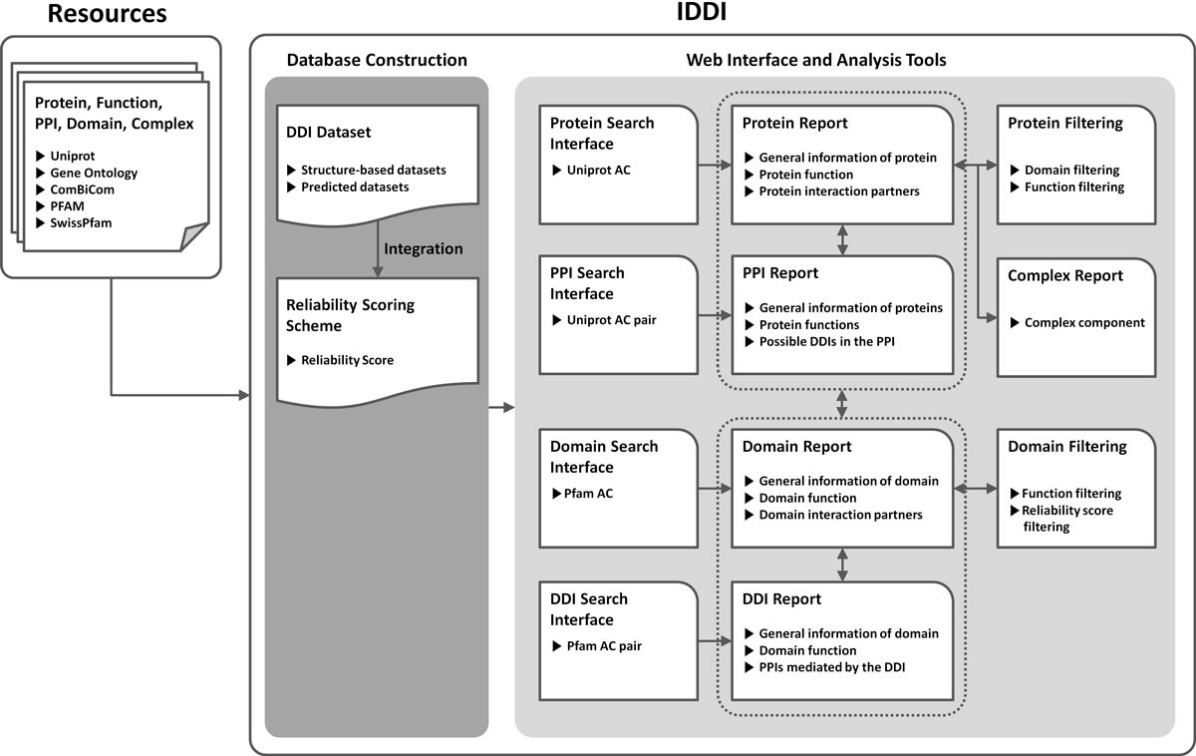
**Schematic illustration of resource collection, database construction and representation of IDDI**.

We constructed a web-based domain-domain interaction analysis interface to provide comprehensive exploration of DDI within protein interaction network. IDDI was constructed on Linux environment and tested for cross-browsing. This system is serviced on tomcat server with Oracle database and web pages were implemented in JAVA and JAVA Server Pages (JSP). Figure 2 presents the system architecture of IDDI. IDDI is composed of three components - a database with an update module, analysis module, and web user interface. Data used in IDDI is imported from each reference database to our database; and our database is semi-automatically updated by the update module. Based on the database information, system provides search result for given query and analysis for given query is executed on analysis module. Web user interface mediates communications between end user and analysis module by user-friendly webpages.

IDDI doesn`t include our new integrated DDI database only but also protein interactions from ComBiCom [28] to grasp the detailed interactions in both domain and protein level. ComBiCom, developed in our group, is the database system providing 257,902 non-redundant binary PPIs and 11,964 protein complexes from 9 experimentally identified PPI databases, which cover the most of publically available PPI information. In order to mapping of domains to their containing protein, SwissPfam available at the Pfam site was used. It provides SWISS-PROT and TrEMBL proteins with their assigned Pfam domains. In addition, we stored protein functional annotations obtained from the Gene Ontology to build a reference set of functional information. An update module is also implemented to semi-automatically update database.

IDDI provides four kinds of searching services: protein search, domain search, PPI search, and DDI search. This searching system is based on PFAM ID and Uniprot accession number for domain and protein classifier, respectively. PPI relationship was searched from ComBiCom, and protein function information is annotated from Gene Ontology. To provide comprehensive searching system, we need to map proteins with their contained domains and SwissPfam was used to map proteins with their corresponding domains. Using this mapping data, IDDI could provide possible DDIs for protein search or possible PPIs for DDI search.

## Results

### Data statistics

**Figure 3:**
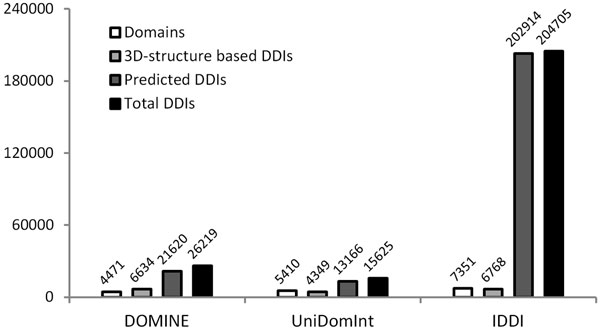
**Data statistics in different DDI databases**.

Our new DDI database currently contains 204,705 unique DDIs between 7,351 distinct Pfam domains. Among DDIs, 6,768 interactions were combined from 3D structure-based datasets and 202,914 interactions were extracted from predicted datasets. It is superior to currently available comprehensive DDI databases such as DOMINE and UniDomInt in a number of both interactions and domains (Figure 3). Massive amount of DDIs are increased due to the employment of the latest released datasets and new datasets. Several DDI datasets such as iPfam, 3DID, InterDom and DIMA show great increased interactions at newly updated releases. Introduction of the new datasets such as PInS, APMM, IPPRI, Top-down, LLZ and TW also provide 47,928 DDIs including 19,609 novel interactions.

### Performance evaluation of reliability scoring scheme in IDDI

**Figure 4:**
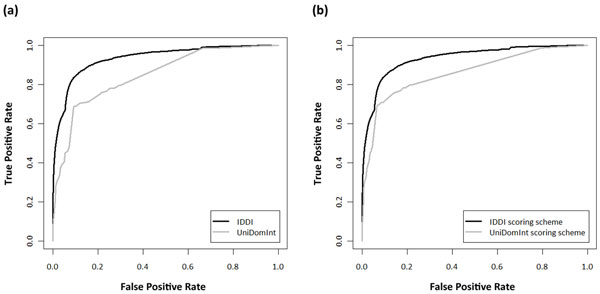
**Performance comparisons of reliability scoring schemes between IDDI and UniDomInt (a) with their own DDI datasets and (b) with the same DDI dataset of IDDI**.

Unlike DOMINE, both IDDI and UniDomInt have linear scoring schemes. Although they considered the confidence of the predicted datasets in common, IDDI reflect the independency of the datasets and the prediction scores of the DDIs additionally. We tried to evaluate the performance of datasets and scoring schemes used in IDDI and UniDomInt with ROC curves (Figure 4). For the sake of fairness, the 3D structure-based DDIs in IDDI were considered as the GSP set to both IDDI and UniDomInt where all of 3D structure-based DDIs in UniDomInt are included in IDDI.

Figure 4(a) shows ROC curves of IDDI and UniDomInt with their own DDI datasets and scoring schemes. The ROC curves demonstrate that IDDI has high true positive rate than UniDomInt at same false positive rate. It indicates IDDI has greater power to filter more reliable DDIs. UniDomInt combines only 8 predicted datasets and the reliability score of UniDomInt is heavily dependent on ME owing to its overwhelming accuracy. It inhibits an accurate measurement of the reliability scores. On the other hand, IDDI include additional predicted datasets including TW, DIPD and Top-down which have as high confidence as ME. It prevents the excessive focus of the reliability scores on a single predicted dataset. For example, interaction between Signal peptide binding domain (PF02978) and SRP19 protein (PF01922), the known DDI searched in iPfam, is found only in the p-value method among 13166 predicted DDIs of UniDomInt and has low reliability score, 0.0548. This score is ranked in the top 87.3% of the total predicted interactions, which means it has high possibility of being false positive. On the other hand, IDDI has additional prediction information for the same DDI from the updated version of InterDom and DIPD, APMM and Top-down, which are not existing datasets in UniDomInt. IDDI's reliability score for this DDI is ranked in top 0.42% of the total predicted interactions and represents high probability of being true positive.

Figure 4(b) shows a comparison between IDDI and UniDomInt`s scoring schemes with same DDI datasets in IDDI. A result reveals that additional factors in our new scoring scheme are efficient enough to filter reliable interactions. UniDomInt considers only the confidence level of the predicted datasets for accessing the reliability score to the each DDI. As a result, comparisons between DDIs found in the same dataset are impossible because all of them receive same scores. It also causes an overestimation problem of the reliability scores. DDIs in a high-confidence dataset are accessed high reliability scores even if they are more likely to false positive because of their low prediction scores.

We tested the average accuracy for reliability score cut-off in IDDI. The result reveals that the cut-off of 0.329 has the highest accuracy, 0.98. For reference, cut-off value that shows 0.90 of accuracy was 0.102 and 21027 DDIs were included within the cut-off value. End user can determine the cut-off value for research purpose and those DDIs which have cut-off value for high accuracy may show more reliable results.

### Comparison of PPI coverage rates

We tried to compare the PPI coverage rate of 3D structure-based DDIs, DOMINE, UniDomInt and IDDI by using binary PPIs in ComBiCom. We defined that the PPI is covered when at least one DDI are found between interacting proteins.

Table 2 shows the number of covered PPI, the number of non-covered PPIs and PPI coverage rate for each DDI data. 3D structure-based DDIs cover only 10.0% of PPIs. On the other hand, IDDI covered 50.4% of PPIs and it is more than twice the coverage rate of DOMINE.

**Table 2:** Comparison of PPI coverage rates in different DDI databases

	3D-structure based only	UniDomInt	DOMINE	IDDI
**PPI with DDI**	25,944	55,788	60,758	129,922
**PPI without DDI**	231,958	202,114	197,144	127,980
**Rate (%)**	10.0	21.6	23.6	50.4

### Functionality of the integrated interaction analysis system

**Figure 5:**
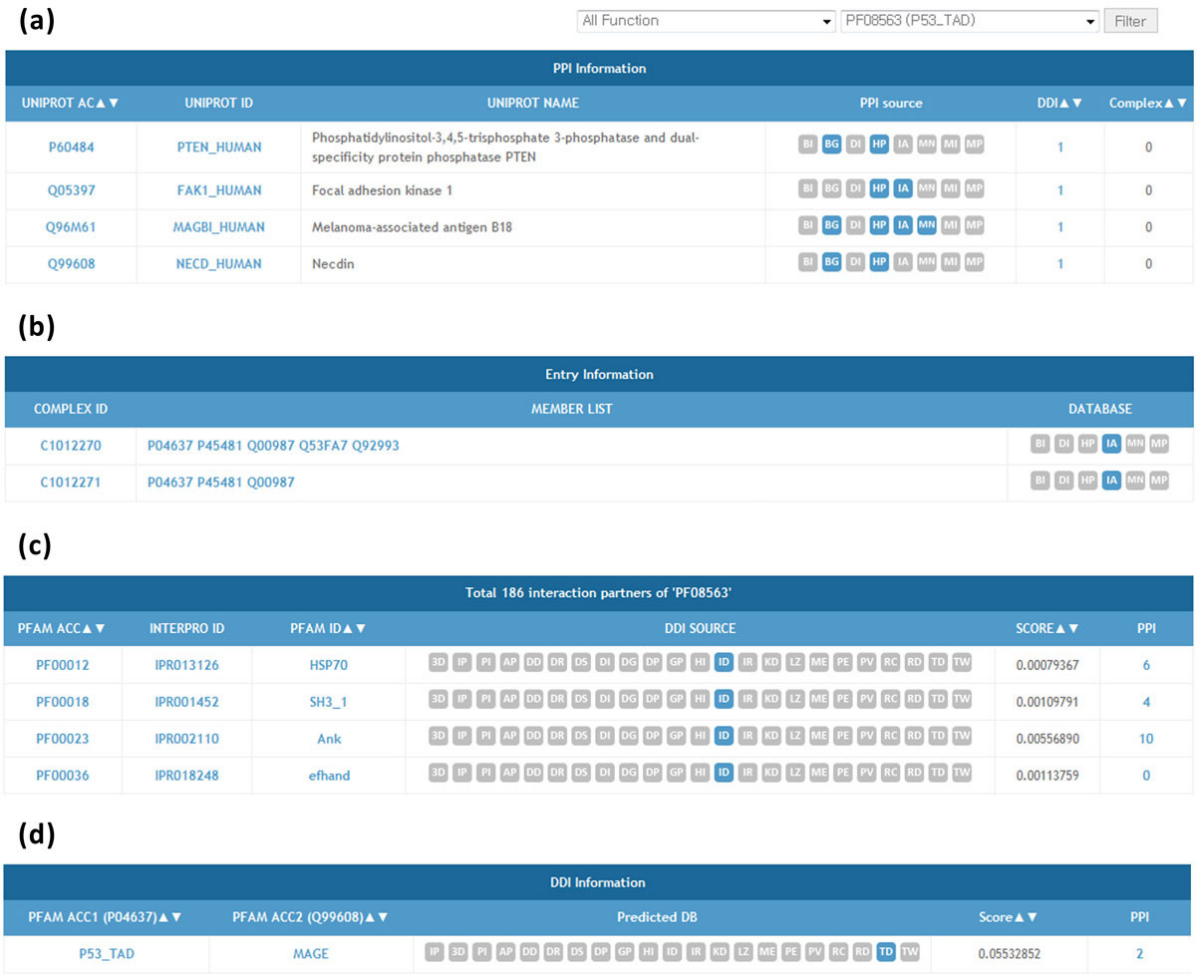
**Example for IDDI functionalities (a) Protein interaction partners of P53 (P04637) having DDI relationship with the P53 transactivation domain (PF08563) (b) Complex information containing P53 and MDM2 (Q00987) (c) Domain interaction partners of P53 transactivation domain (d) DDI information between P53 and Necdin (Q99608)**.

IDDI was constructed to provide comprehensive search on protein or domain to give an insight on detailed interaction. We lay emphasis on easy information access among related proteins, domains, and complexes. The system includes protein, domain, PPI and DDI search system (see Figure 5). First, in protein search, user can check the information of query protein such as protein function referring from Gene Ontology, containing domains from SwissPfam, and known binary PPIs and complexes related to the protein referring from ComBiCom (see Figure 5(a) and 5(b)). Users can easily access to the detailed information about each listed containing domains or PPIs to investigate the working mechanism of the protein. Second, in domain search, DDIs related to the query domain and proteins which contains the domain is provided (see Figure 5(c)). Third, in PPI search, user can search for two proteins to check whether they are known interacting proteins. Also, the system analyzes the domains of each protein and predicts the possible DDIs between two proteins (see Figure 5(d)). Last, in DDI search, given a query of two domains, IDDI checks whether the two domains have DDI relationship and predicts possible PPIs induced from the DDI.

### Example of integrated interaction analysis

IDDI provides comprehensive searching service to explore the relationship of proteins and domains. It can be used for gene selection for study by prioritization of list of proteins with using filtering function. In this section, we provide an example of p53 interacting target analysis. Figure 5(a) and 5(d) illustrates the example of integrated analysis for the specific PPIs and DDIs of p53 protein. Interacting partners for p53 can be searched using protein search and the list of interacting partners are subdivided by domain interaction. Among them, those which have domain interaction with the transactivation domain of p53 can be selected using filtering option (Figure 5(a), only the part of the list is shown here). With this specification of interacting partners, total 11 interacting partners were selected from the 355 partners of p53. The specified DDI can be further investigated by the "DDI" link as shown in Figure 5(d). In this example, as a summary, it shows that the Mage domain of Necdin interact with the transactivation domain of p53. Actually, the interaction mechanism of both domains for the function of two proteins has been turned out by the elaborated experimental works [29]. The investigation can be expanded more with other selected proteins or by tracing the other proteins having Mage domain by using our system. As in this example, our system will enable more sophisticated and efficient investigation about the protein interaction and their function by providing an integrated analysis scheme of DDIs and PPIs.

## Conclusions

We proposed a new unified interaction analysis system, IDDI, which enables the comprehensive analysis of protein and domain interactions with their interconnectivity. Large increase of total DDIs enables high interconnectivity of DDIs and PPIs and an advanced scoring scheme enhances the reliability of integrated DDIs in a substantial amount. Furthermore, IDDI provides a convenient interface to investigate the protein interaction with detail domain interaction. IDDI will be a valuable resource for the in-depth study of interaction mechanism and thereby to derive the functional implication of interacting proteins.

## Competing interests

The authors declare that they have no competing interests.

## Authors' contributions

YK integrated all DDI datasets, analyzed the datasets. BM constructed the web-based analysis system. GSY conceived and supervised this study. YK, BM and GSY wrote the manuscript. All authors read and approved the final manuscript.
